# Symptoms associated with urinary tract infection in nursing home residents: a study among nursing home staff across eight European countries

**DOI:** 10.1016/j.infpip.2026.100563

**Published:** 2026-06-25

**Authors:** A. Chalkidou, V. Antsupova, A.M. Theut, T. Marloth, J. Lykkegaard, M.P. Hansen, N. Sodja, A. Kowalczyk, R. Monfà, L. Jaruseviciene, A. Garcia-Sangenís, C. Llor, A. Bálint, J. Glasa, M. Anastasaki, J.N. Jensen

**Affiliations:** a Research Unit for Antibiotic Stewardship and Implementation, Department of Clinical Microbiology, Copenhagen University Hospital, Herlev and Gentofte, Copenhagen, Denmark; b Research Unit of General Practice, Department of Public Health, University of Southern Denmark, Odense, Denmark; c Health Center Logatec, Logatec, Slovenia; d Department of Family Medicine, Faculty of Medicine, University of Ljubljana, Ljubljana, Slovenia; e Centre for Family and Community Medicine, The Faculty of Health Sciences, The Medical University of Lodz, Poland; f Primary Care Research Institute Jordi Gol (IDIAP), Barcelona, Spain; g Department of Family Medicine, Lithuanian University of Health Sciences, Kaunas, Lithuania; h Institut Català de la Salut, Barcelona, Spain; i Center for General Practice at Aalborg University, Aalborg, Denmark; j Szeged Autumn Nursing Home, Szeged, Hungary; k Department of Health Care Ethics, Faculty of Nursing and Professional Health Studies, Slovak Medical University, Bratislava, Slovakia; l Clinic of Social and Family Medicine, School of Medicine, University of Crete, Heraklion, Greece

**Keywords:** Urinary tract infection, Nursing homes, Elderly, Signs and symptoms, Antibiotic stewardship

## Abstract

Urinary tract infections (UTIs) are common among nursing home (NH) residents and a leading cause of antibiotic use. NH staff members are key in recognizing suspected UTIs based on observed symptoms. This study surveyed 286 staff across eight European countries to identify which symptoms prompt UTI suspicion. While UTI-specific symptoms like painful and frequent urination were commonly cited, non-specific signs such as foul-smelling urine, confusion, and behavioural changes were also frequently reported. These findings reveal substantial cross-country variation and highlight the diagnostic challenges of UTIs in this population, emphasizing the need for improved training in evidence-based symptom recognition.

## Background

Urinary tract infections (UTIs) are the most common indication for antibiotic prescribing among nursing home (NH) residents [1]. However, recent evidence has found that more than half of antibiotic treatments for UTI in this population are potentially unnecessary [1,2]. This unnecessary use of antibiotics not only contributes to the development of antimicrobial resistance (AMR) but also increases the risk of adverse side-effects and complications. AMR is identified by the European Commission as one of the top priority health threats [3]. Within this context, inappropriate antibiotic use for UTIs in NHs appears as a critical aspect of AMR that requires attention [4].

Diagnosing UTIs in NH residents is challenging as these residents often experience a functional decline and cognitive impairments, which can complicate the recognition of specific symptoms [5]. Furthermore, the common presence of asymptomatic bacteriuria – where bacteria are present in the urine without the presence of UTI symptoms – adds to the complexity [6,7]. Among non-catheterized residents in NH facilities, asymptomatic bacteriuria is prevalent in up to 50% of women and 40% of men [8]. Asymptomatic bacteriuria does not require antibiotic treatment [6,7,9], yet routine testing or testing based on false indications often confuses asymptomatic bacteriuria with UTIs, leading to unnecessary antibiotic use [9].

Misconceptions and knowledge gaps among both physicians and NH staff can also play a significant role in the overdiagnosis of UTIs. A recent Delphi study indicated that a prevalent misconception involves attributing non-specific signs and symptoms – such as behavioural changes, agitation, confusion, and cloudy urine – primarily to UTIs [10]. Although there is no gold standard for the diagnostic criteria initiating antibiotics for suspected UTI among NH residents, there is broad agreement that non-specific signs and symptoms alone are insufficient to establish a UTI diagnosis with certainty [11,12].

NH staff members have a central role in supporting the identification of suspected UTI in NH residents as they are typically the first to observe changes in the residents’ health or behaviour. This initial assessment is important as general practitioners affiliated with NHs are influenced to prescribe antibiotics for suspected UTI based on the information provided by the NH staff [6,7]. It has been found that improvement of NH staff’s assessments of signs and symptoms related to UTIs can reduce antibiotic use [9]. However, there is a lack of knowledge of which signs and symptoms are the most frequent assessed by NH staff. This study aimed to explore which signs and symptoms lead NH staff across eight countries to suspect a UTI in NH residents.

## Methods

This study is a part of the European Union (EU)–funded project IMAGINE (*Improving antibiotic use in long term care facilities by infection prevention and control and antibiotic stewardship*) that involves eight countries (Denmark, Greece, Hungary, Lithuania, Poland, Slovakia, Slovenia, and Spain) [13], aiming to reduce inappropriate use of antibiotics by implementing a multi-faceted intervention targeting NH staff. Participating NHs were recruited through national project co-ordinators in each country by information leaflets and information meetings. As a part of the IMAGINE study, a context analysis questionnaire was developed to define quality improvement areas related to infection prevention and antibiotic use in European NHs. The questionnaire was developed during the period December 2022–February 2023. It was developed using materials from the HAPPY PATIENT project [14] and the HALT 3 study [15], with additional input from IMAGINE consortium experts based on their clinical experience. Prior to approval by the IMAGINE consortium, the questionnaire was pilot tested in five NHs in Denmark to assess clarity and feasibility. Minor revisions were made to some questions following the pilot test; however, no adjustments were made to the open-ended question used in the present study.

The questionnaire was translated into local languages by consortium members with expertise in infection prevention and antibiotic use and proficiency in English. Translations were reviewed by country co-ordinators to ensure conceptual equivalence and contextual appropriateness across settings. A formal cross-cultural adaptation process, including back-translation and cognitive testing, was not performed. Subsequently, the questions were inserted into SurveyXact (Rambøll Group A/S, Copenhagen) and through local partners distributed via email to contacts in the participating NHs. These contacts then shared the survey link with NH staff members who were designated as IMAGINE ambassadors. An IMAGINE ambassador was an employee – typically a nurse or a healthcare assistant – at the participating NH working with or responsible for the daily care and hygiene of the residents. The data collection phase was carried out from March to May 2023.

The questionnaire consisted of 19 main questions covering the following domains: care co-ordination, infection prevention, hand hygiene, UTI diagnosis and management, and antimicrobial stewardship. For a detailed view of the questionnaire, refer to appendix file A1.

To evaluate which signs and symptoms prompt NH staff to consider a UTI diagnosis in NH residents without an indwelling urinary catheter, the following open-ended question was constructed: ‘*Which is/are the most important sign(s) that prompt you to consider a urinary tract infection for a nursing home resident without an indwelling urinary catheter?’* allowing respondents to freely express their thoughts without being guided by predetermined response categories related to UTI signs and symptoms.

The analysis of this question involved an initial quantification of each sign or symptom mentioned in the responses, followed by grouping similar signs or symptoms together (for example, all mentions of ‘hematuria’, ‘blood in urine’, ‘bloody urine’, ‘red urine’, and ‘blood clots in urine’ were aggregated under the umbrella term ‘hematuria’). Subsequently, the 10 most stated specific and non-specific signs and symptoms per country were summarized in text format. Symptoms reported by only one respondent within a country were excluded from the country-level summaries.

## Results

A total of 286 NH staff members responded to the questionnaire (Table I). The question about signs and symptoms that prompt a suspicion of a UTI in a resident was mandatory to respond, but five participants did not answer the question (they responded with a dash ‘-’), and two of them responded ‘I don’t know’; therefore, these responses were excluded from the analysis.

**Table I:** Total number of participants who responded to the questionnaire divided by country and profession

Country	DK *N*	GR *N*	HU *N*	LT *N*	PL *N*	SK *N*	SL *N*	ES *N*	Overall *N*
Profession									
Co-ordinating nurse	5	2	9	2	0	36	17	3	74
Nurse	12	4	1	5	3	18	22	4	69
Medical doctor	0	3	0	0	0	1	5	2	11
Care worker with healthcare professional background	46	6	1	7	2	3	9	0	74
Care worker without healthcare professional background	4	1	2	1	0	0	5	0	13
Other^a^	8	5	4	7	0	13	7	1	45
Total	75	21	17	22	5	71	65	10	286

DK, Denmark; GR, Greece; HU, Hungary; LT, Lithuania; PL, Poland; SK, Slovakia; SL, Slovenia; ES, Spain.

a Social worker, nursing home assistant, healthcare assistant, leader of nursing homes, department/division manager, pharmacist, occupational therapist, physiotherapist, administrative worker, and unskilled worker.

Across all countries, both UTI-specific and non-specific symptoms were reported (Table II).

**Table II:** Most cited signs and symptoms of urinary tract infections per country

Denmark (*N* = 75)	%	Greece (*N* = 21)	%	Hungary (*N* = 17)	%	Lithuania (*N* = 22)	%
Confusion	54.7	**Painful urination**	95.2	**Painful urination**	100.0	Foul smell of urine or odour change	59.1
Foul-smelling urine or odour change	42.7	Fever	81.0	Fever	94.1	Changed colour of urine	40.9
Behaviour change	37.3	Haematuria	81.0	**Frequent urination or increased urge**	70.6	**Painful urination**	40.9
**Frequent urination or increased urge**	34.7	**Frequent urination or increased urge**	66.7	**Low abdominal pain**	47.1	Fever	40.9
**Burning sensation**	29.3	Difficult urination	47.6	Foul-smelling urine or odour change	35.3	**Frequent urination or increased urge**	22.7
Fever	26.7	General malaise	33.3	Cloudy urine/opalescent urine	23.5		
**Painful urination**	18.7	Pain in the lower back	28.6	Urge	17.6		
Cloudy urine	14.7	Foul smell of urine or odour change	28.6	Lower back pain	17.6		
Haematuria	6.7	**Low abdominal pain**	23.8	Loss of appetite	17.6		
Slurred speech	6.7	Poor condition	23.8	Haematuria	17.6		

Poland (*N* = 5)	%	Slovakia (*N* = 71)	%	Slovenia (*N* = 65)	%	Spain (*N* = 10)	%

**Frequent urination**	40	**Painful urination**	56.3	Foul smell of urine or odour change	41.5	Fever	60
**Painful urination**	40	**Burning sensation**	56.3	Confusion	38.5	Disorientation	40
**Burning sensation**	40	**Frequent urination or increased urge**	50.7	Dark urine or change of colour	38.5	General malaise or deterioration	40
Haematuria	40	Fever	42.3	Fever	38.5	**Painful urination**	40
		Low abdominal pain	22.5	**Burning sensation**	20.0	**Frequent urination or increased urge**	30
		Foul smell of urine or odour change	19.7	Behaviour change	16.9	Aggressiveness or irritability	30
		Haematuria	15.5	Restlessness	12.3	Behaviour change	20
		Change of colour of the urine	15.5	Low abdominal pain	10.8	Foul smell of urine or odour change	20
		Cloudy or concentrated urine	11.3	Cloudy urine or change of urine density	10.8	Haematuria	20
		Lower back pain	8.5	**Frequent urination**	9.2	**Low abdominal pain**	20

UTI-specific symptoms [11] are highlighted in bold.

UTI, urinary tract infection.

The most frequently cited UTI-specific symptoms included *painful urination* (reported in all eight countries) and *frequent urination* (seven countries). Other UTI-specific symptoms such as *low abdominal pain* and *lower back pain* were mentioned in several countries.

Non-specific symptoms were also commonly cited. *Foul-smelling urine* was reported by NH staff in all countries except Poland. Frequently mentioned were also f*ever or elevated temperature* (six countries) and *haematuria* (six countries). *Behavioural changes*, including *confusion*, *restlessness*, *aggressiveness*, and *disorientation*, were frequently noted, particularly in Denmark, Slovenia, and Spain. *Cloudy urine* or *changes in urine colour or consistency* was also frequently mentioned across countries.

In addition to the typical symptoms that are attributed to UTI, some NH staff mentioned non-clinical signs such as fall episodes, lethargy, and vomiting as indications of UTI. These signs and symptoms were not commonly mentioned, and they are not considered relevant to a UTI diagnosis. These can be found in the appendix file A2.

## Discussion

### Discussion of main findings

This study found that the most cited signs and symptoms of UTI varied largely across countries. NH staff frequently associated both UTI-specific symptoms – such as painful urination – and frequent urination with UTI. Additionally, non-specific symptoms like foul-smelling urine, confusion, and behavioural changes, which can be associated with a variety of infections or health conditions, were also prominent in the study sample, often being the most frequently mentioned symptoms in several countries.

The findings are in line with other studies that found that non-specific symptoms play a significant role in the diagnosis of UTI [5,8,16,17]. Also, a recent study found that over half of the antibiotics given for suspected UTI in NHs across the eight EU countries were potentially unnecessary as the specific symptoms for having a UTI were not met [18]. It could be speculated that potentially unnecessary use for suspected UTI may be driven by lack of a definitive gold standard for diagnosing UTI.

Examining the different signs and symptoms that prompt NH staff to suspect a UTI diagnosis reveals the challenging nature of diagnosing UTIs in older adults and especially older adults with cognitive impairments [19]. The observed variation in reported signs and symptoms may reflect differences in training, education, and clinical experience among NH staff. The staff members who have daily contact with residents, assisting them with day-to-day activities, may not necessarily have a healthcare education. Despite this, they are often the first to notice changes in the behaviour of the residents [13] and subsequently reach out to a nurse, who then contacts a physician [14]. Providing targeted education to all levels of NH staff may improve the overall effectiveness of UTI diagnosis and management in older adults [20].

Differences in the specificity and details of symptom descriptions may also reflect variation in clinical language, documentation practices, or national training standards. Such differences could have implications for communication in cross-border healthcare settings and efforts to harmonize diagnostic criteria. It could be hypothesized that countries that listed a broader range of symptoms may be working within more detailed clinical guidelines or structured protocols, whereas shorter symptom lists might reflect less formalized approaches or a greater reliance on laboratory confirmation.

The findings raise the question of whether a standardized EU-wide symptom checklist for suspected UTIs in older adults, combined with targeted training and education of NH staff, would reduce diagnostic variability, or whether flexibility is needed to accommodate diverse clinical and cultural contexts.

Future research could explore how these diagnostic cultures, as well as differences in training and educational approaches, influence antibiotic prescribing and patient outcomes.

### Strengths and limitations

This study has some strengths and limitations. The major strength of our study lies in the fact that we invited NH staff to freely express which signs and symptoms lead them to suspect a UTI diagnosis, without imposing predetermined response categories. The open-ended nature of the question allowed us to capture a wide range of responses, including non-specific signs and behavioural changes, that may not have been identified using a predefined multiple-choice question. However, a limitation of this approach is that a large number of open-ended responses required translation from multiple national languages into English. These translations were performed by the first author and reviewed by country co-ordinators fluent in both English and the original language prior to analysis. Analysing the open-ended responses posed challenges due to substantial variation in terminology used to describe similar symptoms across languages and respondents (e.g. multiple expressions referring to haematuria), requiring interpretive grouping of responses and limiting the ability to calculate precise symptom frequencies. The number of responses varied substantially between countries, and findings from countries with very few responses (Poland, *N* = 5; Spain, *N* = 10) should therefore be interpreted with caution as the dominant signs and symptoms identified may not be representative and could change with a larger sample. As the data were self-reported by NH staff, there is a risk of response bias which may have influenced how signs and symptoms were reported. Although rare, a small number of respondents indicated uncertainty regarding which symptoms prompt suspicion of UTI, and this may indicate a need for continued education in this area. Lastly, we do not know whether all participating NHs are represented in the survey, and we do not have the total number of participants that the invitation to the survey reached. Thus, we were not able to calculate a response rate for our questionnaire.

In conclusion, although there were some differences in the frequency of responses across countries, the pattern of relying on both specific and non-specific signs was consistent. This highlights the ongoing challenge of diagnosing UTIs in NH settings and suggests a need for targeted educational interventions.

## CRediT authorship contribution statement

**A. Chalkidou:** Writing – review & editing, Writing – original draft, Validation, Project administration, Methodology, Formal analysis, Data curation, Conceptualization. **V. Antsupova:** Writing – review & editing, Validation, Supervision. **A.M. Theut:** Writing – review & editing, Validation, Investigation. **T. Marloth:** Writing – review & editing, Investigation. **J. Lykkegaard:** Writing – review & editing, Investigation. **M.P. Hansen:** Writing – review & editing, Investigation. **N. Sodja:** Writing – review & editing, Investigation. **A. Kowalczyk:** Writing – review & editing, Investigation. **R. Monfà:** Writing – review & editing, Investigation. **L. Jaruseviciene:** Writing – review & editing, Investigation. **A. Garcia-Sangenís:** Writing – review & editing, Investigation. **C. Llor:** Writing – review & editing, Investigation. **A. Bálint:** Writing – review & editing, Investigation. **J. Glasa:** Writing – review & editing, Investigation. **M. Anastasaki:** Writing – review & editing, Investigation. **J.N. Jensen:** Writing – review & editing, Writing – original draft, Supervision, Project administration, Methodology, Investigation, Data curation, Conceptualization.

## Ethics statement

The study protocol was approved in Spain, the co-ordinating country, by the Ethics Committee of IDIAP Jordi Gol, Institute of Research in Primary Health Care (ref. 23/080-P) on 12^th^ July 2023.

## Funding sources

The IMAGINE project was funded by the EU4Health Program Project (Grant Agreement: 101079838).

## Conflict of interest statement

The authors declare that there is no conflict of interest.
