# Supplementary material for: Symptoms associated with urinary tract infection in nursing home residents: a study among nursing home staff across eight European countries
Source: Infect Prev Pract. 2026 Jun 25;8(3):100563. doi: 10.1016/j.infpip.2026.100563 (PMC13400360; doi:10.1016/j.infpip.2026.100563)
Supplement: Supplementary file 1 [file mmc1.docx]

**Context Analysis questionnaire**

**Which country participating in the IMAGINE project do you represent?**

1. Denmark
2. Greece
3. Hungary
4. Lithuania
5. Poland
6. Slovakia
7. Slovenia
8. Spain

**What is your profession?**

1. Coordinating nurse
2. Nurse
3. Medical doctor
4. Care worker (with health care professional background)
5. Care worker (without health care professional background)
6. Other (please specify):

## Coordination of care

1. **Which healthcare persons are typically tasked to assist with daily personal hygiene and/or toilet visits? (select all that may apply)**
2. Care workers (with health care professional background)
3. Care workers (without health care professional background)
4. Nurses
5. Other (please specify):
6. **To what extent do the residents’ relatives typically assist with daily personal hygiene and/or toilet visits?**
   1. To a very large or large extent
   2. To some extent
   3. To little or no extent
   4. I don’t know

## Infection prevention and control

1. **Which of the following elements are present in your nursing home? (select all that may apply)**
   1. Guidelines for the management of urinary tract infections
   2. Guidelines for the management of indwelling urinary catheters
   3. Posters/infographics in the nursing home with information about hygiene practices
   4. Registration of residents infected with (multi-)resistant microorganisms
   5. Registration of residents infected with any type of microorganisms
   6. A person designated for reporting and managing infection outbreaks
   7. A person designated for infection prevention and hygiene
   8. Isolation or additional precautions for residents infected with a highly contagious disease or (multi-)resistant microorganisms
   9. None of the above
   10. I don’t know
   11. Other (please specify):

**3a. If there are guidelines for the management of urinary tract infections present in your nursing home (select yes or no):**

- 1. They are accessible to me – Yes/No/I don’t know
  2. They are easy to understand – Yes/No/I don’t know
  3. They are useful to me – Yes/No/I don’t know
  4. They are insufficient – Yes/No/I don’t know

**3b. If there are guidelines for the management of indwelling urinary catheters present in your nursing home (select yes or no):**

- 1. They are accessible to me – Yes/No/I don’t know
  2. They are easy to understand – Yes/No/I don’t know
  3. They are useful to me – Yes/No/I don’t know
  4. They are insufficient – Yes/No/I don’t know

1. **How often do you receive a practical training in infection prevention (e.g. cleaning, personal hygiene, toilet visits)?**
   1. Only during introduction
   2. Frequently
   3. Occasionally
   4. Rarely
   5. Never
2. **Are there any challenges in your daily practice that you would like to overcome when it comes to infection prevention? (open ended question)**

## Hand hygiene

1. **How often do you receive practical training in hand hygiene?**
   1. Only during introduction
   2. Frequently
   3. Occasionally
   4. Rarely
   5. Never
2. **Are there hand wash sinks in the resident rooms?**
   1. Yes, in all the resident rooms
   2. In some of the resident rooms
   3. No, in none of the resident rooms
3. **Is there alcohol rub solution available in the resident rooms?**
   1. Yes, in all the resident rooms
   2. In some of the resident rooms
   3. No, in none of the resident rooms
4. **In your nursing home, is there an infographic or a poster about hand hygiene visible close to the hand wash sinks?**
   1. Yes
   2. No

## Infection diagnosis and management – Urinary tract infections (without urinary indwelling catheter)

1. **Which is/are the most important sign(s) that prompts you to consider a urinary tract infection for the nursing home resident without a urinary indwelling catheter? (open ended question)**
2. **What do you usually do first when you suspect a urinary tract infection in a nursing home resident? (select all that may apply)**
   1. Ask the resident about their symptoms
   2. Perform a urine dipstick
   3. Take a urine sample to the doctor for urine culture
   4. Provide antibiotics as soon as possible
   5. Exclude other possible diagnoses before other actions
   6. Involve a doctor
   7. Wait and monitor a possible progression of symptoms
   8. Improve areas related to infection prevention (e.g. toilet visits, hydration, residual urine)
   9. Other (please specify):
3. **From your point of view to what extent can urinary tract infections be avoided in the elderly who live in nursing homes?**
   1. To a very large or large extent
   2. To some extent
   3. To little or no extent
   4. I don’t know
4. **From your point of view to what extent can urinary tract infections be prevented in your nursing home by utilizing infection prevention?**
   1. To a very large or large extent
   2. To some extent
   3. To little or no extent
   4. I don’t know

## Infection diagnosis and management – Urinary tract infections (with urinary indwelling catheter)

1. **In relation to residents with a urinary indwelling catheter, which of the following elements are present in your nursing home? (Select all that may apply)**
   1. List of indications about when to insert an indwelling catheter (indications)
   2. Protocols for handling a resident with an indwelling catheter
   3. Designated persons to handle residents with an indwelling urinary catheter
   4. None of the above
   5. I don’t know
2. **How often do you receive in-house training for handling residents with an indwelling urinary catheter?**
   1. Only during introduction
   2. Frequently
   3. Occasionally
   4. Rarely
   5. Never

## Antimicrobial stewardship (guidelines and management of antibiotics)

1. **Who prescribes antibiotics in the nursing home? (select all that may apply)**
2. A medical doctor, attached to the nursing home
3. Any medical doctor, not attached to the nursing home
4. Medical staff employed at the nursing home other than medical doctors
5. Other persons
6. Antibiotics can be dispensed without prescription
7. **Are there written guidelines for appropriate antimicrobial use (good practice) in your nursing home?**
   1. Yes
   2. No
   3. I don’t know

**17a. If there are guidelines for appropriate antimicrobial use (good practice) present in your nursing home (select yes or no):**

1. They are accessible to me – Yes/No/I don’t know
2. They are easy to understand – Yes/No/I don’t know
3. They are useful to me – Yes/No/I don’t know
4. They are insufficient – Yes/No/I don’t know
5. **From your point of view to what extent is antimicrobial resistance a problem in your nursing home?**
   1. To a very large or large extent
   2. To some extent
   3. To little or no extent
   4. I don’t know
6. **How common is prophylactic antibiotic treatment for urinary tract infections in your nursing home?**
   1. Very common
   2. Common
   3. Not so common
   4. I don’t know
