# Supplementary material for: Symptoms associated with urinary tract infection in nursing home residents: a study among nursing home staff across eight European countries
Source: Infect Prev Pract. 2026 Jun 25;8(3):100563. doi: 10.1016/j.infpip.2026.100563 (PMC13400360; doi:10.1016/j.infpip.2026.100563)
Supplement: Supplementary file 2 [file mmc2.docx]

**Supplementary file S2.**

Not-typical signs and symptoms of UTI mentioned by the participants

In addition to the typical symptoms that are attributed to UTI, some nursing home staff mentioned signs and symptoms not entirely relevant to a UTI diagnosis. Although these symptoms were not commonly mentioned in our sample, they are worth noting for a comprehensive understanding of the factors influencing UTI diagnosis in a nursing home facility. These include the following: fall episodes and injuries, lethargy or an increased need for bed rest, feelings of nausea, vomiting, or a reduced appetite, poor food quality, rejection of food or water, dehydration, fear of urination, leaning on one side, difficulty emptying the bladder, skin inflammation, restricted movement, hyperreactivity, and the presence of a urinary catheter.
